# The Effectiveness of an Augmented Reality-Based Early Intervention Program Using Interactive Games to Enhance Eye Contact as a Nonverbal Communication Skill in Children with Autism: A Single-Case Experimental Design

**DOI:** 10.3390/jintelligence14040064

**Published:** 2026-04-10

**Authors:** Shoeb Saleh, Rommel AlAli

**Affiliations:** The National Research Centre for Giftedness and Creativity, King Faisal University, Al-Ahsa 31982, Saudi Arabia; sgsaleh@kfu.edu.sa

**Keywords:** autism spectrum disorder, augmented reality, non-verbal communication, eye contact, early intervention, single-case design, Saudi Arabia

## Abstract

Children with Autism Spectrum Disorder (ASD) frequently exhibit marked impairments in nonverbal communication, particularly in eye contact, which serves as a foundational element for social interaction and relational development. This study evaluated the effectiveness of an early intervention program utilizing interactive games supported by Augmented Reality (AR) technology to enhance eye contact behaviors, specifically initiation and maintenance, in children with autism. Using a multiple baseline across participants single-case experimental design, four boys (aged 5–7 years) diagnosed with ASD participated in an 8-week intervention at a specialized center in Saudi Arabia. The intervention featured tablet-based, gamified AR tasks incorporating real-time visual feedback, graduated difficulty levels, and reinforcement mechanisms designed to elicit social gaze and sustained eye contact. Eye contact duration and frequency were measured during structured social interactions via systematic direct observation. The results demonstrated significant improvements across all participants, with the mean duration of eye contact increasing from a baseline of 2.0 s to 5.8 s post-intervention. Visual analysis revealed robust treatment effects, further supported by substantial Tau-U effect sizes (range = 0.89–0.96; M = 0.93). Follow-up data collected three weeks post-intervention confirmed the maintenance of gains for three of the four participants. These findings suggest that AR-based interventions provide an effective and culturally responsive approach for enhancing specific nonverbal communication behaviors among children with autism in Middle Eastern contexts. Implications for clinical practice and directions for future research are discussed.

## 1. Introduction

Autism spectrum disorder (ASD) is one of the most common neurodevelopmental disorders, defined by persistent deficits in social communication and interaction, accompanied by restricted, repetitive patterns of behavior, interests or activities (American Psychiatric Association, 2022, p. 53). In that regard, a recent epidemiologic investigation within the Kingdom of Saudi Arabia found an increasing prevalence of autism diagnosis, 5 in every 100 children, and the current estimates are on par with those globally, where it is approximately 1 in every 100 children (Alnemary et al., 2017, p. 890). The increasing awareness of autism has led to a great increase in specialized education and treatment services in all regions of Saudi Arabia, resulting in the setting up of dedicated autism centers such as Al-Dekheil Council for Educational Affairs, Khadija Bint Khuwaylid Center for Autism—Jazan.

One of the core deficits encountered in children with ASD involves disturbances in non-verbal communication, specifically characterized by a lack of eye contact, mutual attention, and shared social interest (Mundy et al., 1994; Mundy & Willoughby, 1996). These behaviors are considered a hallmark of the disorder with critical implications for social learning and development (Bruinsma et al., 2004). While these foundational deficits have been well-documented for decades, recent neuroimaging research has further elucidated the underlying neural correlates of atypical eye contact in social contexts (Hirsch et al., 2022). Gaze remains a critical underpinning of human social interaction, as it facilitates emotional connection and the coordination of joint attention, a process that begins to diverge in early development for children with ASD (Thorsson et al., 2024). Furthermore, these atypicalities often lead individuals to redirect their attention away from facial stimuli toward non-social environmental objects (Freeth et al., 2013).

Theoretical models concerning social attention in Autism Spectrum Disorder (ASD) suggest an early-onset deficit in orienting attention toward faces and social stimuli, which adversely affects the development of nonverbal communication skills, particularly eye contact. These models posit that individuals with ASD exhibit a reduced preference for social stimuli compared to non-social objects, leading to limited opportunities for spontaneous social learning (Dawson et al., 2005). Furthermore, other frameworks indicate that difficulties in initiating and sustaining visual fixation are linked to impairments in social information processing rather than mere visual perception (Klin et al., 2002). Consequently, the literature emphasizes that interventions utilizing structured reinforcement and immediate feedback, particularly those supported by emerging technologies, can effectively redirect attention toward facial stimuli and enhance nonverbal communicative behaviors (Chevallier et al., 2012).

### 1.1. Statement of the Problem

Historically, intervention strategies aimed at remediating visual communicative deficits in children with autism have overwhelmingly centered on behaviorally based methodologies like (discrete trials training, naturalistic behavioral interventions, social skills training). Although these proven treatments are effective, they are typically delivered in a highly labor-intensive one-on-one format, necessitate extensive professional training for delivery, and rely on substantial family and provider time commitments. Additionally, traditional methods may not take full advantage of the technological enjoyment motivations often observed in children with ASD, who commonly exhibit pronounced interest in digital media and screen-based activities.

A critical distinction must be made between Virtual Reality (VR) and Augmented Reality (AR). While VR provides a fully immersive experience that replaces the physical world with a simulated environment, it may occasionally lead to difficulties in generalizing skills to real-world social contexts. In contrast, AR functions by superimposing digital information onto the user’s actual environment, thereby retaining ecological validity (Chen et al., 2020). This unique property allows children to engage with a real human partner while simultaneously receiving digital visual prompts, making AR a more suitable paradigm for addressing social-visual deficits in situ.

In spite of increasingly international evidence for the use of AR applications in the autism field, there was still limited evidence. Most research has been carried out in Western countries, where cultural adaptation or linguistic considerations might jeopardize application to a global audience. In addition, although AR has been used to teach a number of academic skills to children with autism or children with visual impairments (Shepley et al., 2016; Fuentes et al., 2025). While Virtual Reality (VR) replaces the physical environment with a digital one, Augmented Reality (AR) superimposes digital information onto the actual environment, preserving ecological validity (Chen et al., 2020). This distinction is vital because impaired eye contact, a key manifestation of deficits in non-verbal communicative behaviors (DSM-5-TR, Criterion A.2), is best addressed in settings where the child remains connected to a real-world social partner. This is an important research gap, especially in light of the fact that impaired eye contact constitutes both a diagnostic characteristic and a functional impairment in autism.

### 1.2. Purpose of the Study

This was the critical gap that the current study aimed to address by objectively examining the efficacy of a culturally adapted AR-based intervention program in Arabic for ameliorating non-verbal communication behaviors in young children with autism within Saudi Arabia. This study specifically intended to investigate whether an 8-week early intervention that involved tablet games supported by AR design could promote meaningful changes in eye gaze duration and frequency of children enrolled at the Khadija Bint Khuwaylid Center for Autism in Jazan. Using a strict single-case experimental design for multiple participants, the purpose of this study was to delineate functional relationships between the AR intervention and target behaviors by demonstrating response patterns across individuals and maintenance of treatment effects.

### 1.3. Literature Review

#### 1.3.1. Non-Verbal Communication Deficits and Neural Correlates of Gaze in ASD

The origin and the mechanisms of non-verbal communication dysfunctions observed in ASD have long been under a critical evaluation in the theoretical and experimental work of many researchers from different fields (Chung & Son, 2020; Girault, 2025). Current neurocognitive models suggest that the unusual patterns of visual attention often observed in ASD are associated with altered bottom-up perception alongside atypical top-down control processes (Chita-Tegmark, 2016, p. 760). Neuroimaging evidence has reported structural and functional differences in brain regions implicated in social visual processing, such as the superior temporal sulcus, amygdala and fusiform face area that may contribute to diminished attention to faces and eyes among individuals with autism (Hirsch et al., 2022, p. 8).

From a developmental standpoint, the eye avoidance account proposes that reduced eye contact in autism develops early in postnatal life and is a reaction to atypical arousal or stress from looking others directly in the eyes, possibly as a form of self-regulation rather than merely dismissing social stimuli (Tanaka & Sung, 2016; p. 98). This conceptualization has critical design implications, indicating that effective strategies need to strike an appropriate balance between eye contact practice opportunities and participant comfort and stress reduction. Other theoretical narratives, on the other hand, emphasize social motivational impairments, suggesting that people with autism are less inclined to gain an intrinsic reward from engaging in social interaction and, as such, invest less attention to social cues (faces and eyes) (Chevallier et al., 2012, p. 231).

Regardless of the mechanisms involved, the functional ramifications of disrupted non-verbal communication for individuals with autism are great and well established. Eye contact and JA deficits predict long-term outcomes in many areas such as language, achievement and social relationship quality (Chawarska et al., 2013, p. 1566). These results highlight the crucial role of early intervention in non-verbal communication as a precursor to overall developmental growth.

#### 1.3.2. Traditional Interventions for Non-Verbal Communication in Autism

Traditional behavior-based interventions to teach eye contact to children with autism have utilized applied behavior analysis (ABA) in the form of systematic prompting, reinforcement, and fading procedures to increase looking at someone or something for a specified number of seconds. Discrete trial training methods disentangle eye contact into component behaviors and offer multiple repetitions of trials while providing immediate reinforcement for correct responding (Smith, 2001, p. 90). As Bokhari et al. (2007) and Yaruss and Quesal (2002) have shown, however, intensive social skills treatments can lead to increases in eye contact, but there may be questions about the utter authenticity of “trained” behaviors as they carry over from training contexts to more naturalistic social environments.

More recent techniques concentrate on naturalistic developmental behavioral interventions (NDBIs) that situate eye gaze practice in functional, child-initiated activities in natural settings (Schreibman et al., 2015, p. 3). Other NDBI programs, such as the Early Start Denver Model, Joint Attention Symbolic Plan Engagement and Regulation (JASPER)3, have demonstrated initial evidence for targeting social communication, such as joint attention and eye contact, with a possible added value of engendering more spontaneous use of these skills in context (Dawson et al., 2010, p. 17). Nonetheless, these methods usually entail rigorous clinician training and delivery schedules that are intense and potentially infeasible for access, especially in a resource-limited context.

#### 1.3.3. Augmented Reality Technology in Special Education

Augmented reality is a relatively new technology, and its use in the area of education is growing, including for students with disabilities. AR, as defined through computer-mediated sensory information overlaid in real-time onto physical reality (Azuma, 1997, p. 356), creates hybrid experiences that are connected to the real world and offer digital support and augmentation. In contrast, virtual reality takes the user into entirely computer-generated worlds that only substitute for, rather than augmenting, physical surroundings.

The distinctive features of AR are well balanced in relation to learning traits and needs, especially on the part of students with autism. First, AR systems can deliver instant visual feedback and reinforcement on specific behaviors to assist with the direct behavior-consequence contingencies needed for the learning of a large number of people with ASD (Chen et al., 2020, p. 5). Second, AR programs have the capacity to systematically manipulate stimulus complexity and slowly fade in social demand (while still being predictable and structured), which could attenuate anxiety for learners with autism. Third, through embedding digital in real-world contexts, AR might be able to better generalize the learning from a training setting into everyday life as compared with pure virtual reality McMahon et al., 2016, p. 102).

Recent systematic reviews have integrated knowledge about AR treatments for persons with autism in a range of skill areas. Lorenzo et al. (2019, p. 1) were able to locate a total of 25 studies investigating the use of AR for autism, with positive results in areas such as daily living skills, academic success and social interaction. The authors state that there was substantial methodological variation and recommend better research types to validate evidence-based practice. Likewise, Newbutt et al. (2022) scoping review of this topic had a small data set. Compiling 49 studies on virtual and augmented reality social skills interventions for ASD, found technology acceptance and preliminary efficacy to be promising; however, large-scale RCTs are still limited in number, and there is a significant gender imbalance in the literature.

#### 1.3.4. AR Interventions for Non-Verbal Communication and Eye Contact

Although the general AR literature for ASD includes numerous intervention targets, a few studies have investigated AR applications specifically developed to improve non-verbal communication behaviors, eye contact and face-directed gaze. These studies utilized a variety of technological platforms, ranging from tablet-based AR applications to wearable smart-glasses that include onboard coaching systems. The theoretical basis of AR-based eye contact interventions often relies on the technology’s ability to offer immediate visual information and feedback that guides focus back on faces and is also reinforced through gamification elements, rewarding participation.

The seminal study by Liu et al. (2017, p. 3) for a pilot that tested the feasibility and early efficacy of wearable AR smart glasses (Google Glass) running the “Empowered Brain” Face2Face app to teach eye contact with children and youths on the autism spectrum. The application detected the faces of humans in front of the wearer, tracked their gaze, and then offered multimodal signals (to see on interaction partners’ faces) that guided visual attention towards their interactants. In a single-subject case study of a 13-year-old child with ASD, the intervention was linked to improvements on the social motivation and social cognition subscales of the Social Responsiveness Scale-2 (SRS-2) after 16 ten-minute intervention sessions over three weeks (p. 7). The study showed the feasibility of a teacher-facilitated AR intervention in actual school settings, though a one-subject design was not generalizable.

Inspired by this work, Keshav et al. (2017, p. 1) undertook a larger feasibility trial of the same AR smart-glasses system with 14 children (aged 6–17 yrs) who have autism. The participants completed, on average, 10.5 sessions at home over six weeks. Findings revealed that the intervention was well-tolerated and free from negative side effects, with some evidence of advances in social behaviors and eye contact frequency based on parent report assessments. However, no objective behavioral measures and control comparisons were made; thus, conclusions about treatment effectiveness were compromised.

More recently, Chen et al. (2020, p. 7) developed and evaluated an AR tablet app with 3D animated characters to motivate children’s ideal eye gaze in the form of a game with increasing play levels. The authors used a multiple baseline design across participants with the three youths, 6 to 8 years of age, diagnosed with autism and recorded large increases in duration of eye contact during both directed play activities, with effect sizes (PND) greater than 80% for all three participants. Importantly, gains were generalized to untrained social partners and maintenance data collected over 3 weeks post-intervention indicated that improvements were retained.

#### 1.3.5. Research Gaps and Cultural Considerations

In accordance with the DSM-5-TR’s emphasis on cultural considerations, it is noted that while eye contact is a universal diagnostic marker, its social frequency and intensity may vary by culture. In the Saudi context, the intervention utilized culturally familiar icons like local flora to ensure that the technological interface did not conflict with the child’s environmental expectations.

Despite growing evidence in favor of AR intervention for autism, there are important gaps in the literature. The first is that most studies have been carried out with the English-speaking population living in Western societies, mainly the United States and some European countries. This geographic focus raises issues about the cultural relevance and suitability of current AR applications if transferred to non-Western cultures with different social communication conduct and languages. For instance, cultural differences in the social significance and appropriate level of direct eye contact are reported to exist across cultures, with some Middle Eastern Cultures regarding prolonged direct gaze as a potential sign of disrespect in some situations (McCarthy et al., 2006, p. 1039). These cultural aspects highlight the need for careful tailoring of intervention protocols and measurement when approaches targeted at AR eye contact are deployed to very different contexts around the world.

Second, limited research has addressed AR applications in the Saudi context or the context of other Arab Gulf countries, although substantial amounts of funding have been proposed and invested in educational technology infrastructure, with an observable high prevalence of autism. Studies by Alnemary et al. (2017, p. 1392) have reported substantial disparities in the use of evidence-based practice for children with autism spectrum disorder in Saudi Arabia, where fewer specialized interventions are available beyond applied behavior analysis programs. The current study fills this gap by developing and testing a culturally tailored AR intervention in Arabic, implemented within an autism center in Saudi Arabia, thus providing much-needed evidence for technology-enhanced intervention effectiveness within the Middle East.

Third, the field of AR autism research to date has been fraught with serious methodological problems, such as high representation of case studies and small pilot investigations, scarce implementation of systematic single-case experimental designs that would allow causal inference, inadequate follow-up and generalization assessment, and lack of reporting on procedural integrity and interobserver agreement data. The present study meets these methodological deficiencies by using a strict multiple baselines across participants design, including prolonged baseline phases, systematic control of intervention integrity, and follow-up measures examining maintenance effects.

### 1.4. Research Questions

The study was guided by the following research questions:

1. To what extent does an early intervention program based on interactive games supported by augmented reality technology improve the duration of eye contact during structured social interactions for children with autism at the Khadija Bint Khuwaylid Center in Jazan, Saudi Arabia?
2. To what extent does an early intervention program based on interactive games supported by augmented reality technology improve the frequency of eye contact initiations during structured social interactions for children with autism at the Khadija Bint Khuwaylid Center in Jazan, Saudi Arabia?
3. Do improvements in non-verbal communication behaviors achieved through AR-based intervention maintain for three weeks following program completion?
4. Are there individual differences in participant responsiveness to the AR-based intervention, and what participant characteristics might predict differential treatment outcomes?

### 1.5. Research Hypotheses

Based on theoretical frameworks and empirical evidence from prior AR intervention research, the following directional hypotheses were formulated:

**H_1_.**  *Participants will demonstrate statistically and clinically significant increases in mean eye contact duration (measured in seconds) from baseline to intervention phases, with effect sizes exceeding Tau-U = 0.80.*

**H_2_.**  *Participants will demonstrate statistically and clinically significant increases in eye contact initiation frequency (measured as the number of spontaneous eye contact instances per 5 min observation) from baseline to intervention phases.*

**H_3_.**  *Non-verbal communication improvements observed during the intervention phase will be maintained at levels significantly above baseline during follow-up assessment periods three weeks post-intervention.*

**H_4_.**  *Participants with higher baseline receptive language abilities and lower baseline autism symptom severity will demonstrate larger magnitude improvements in non-verbal communication behaviors.*

## 2. Methods

### 2.1. Research Design

A single-case experimental design (SCED), specifically a multiple baseline across participants design, was used to investigate the functional relation between AR-based intervention for children with autism and non-verbal communication behavior. Single-case designs are recognized as valid and stringent methods of intervention research in autism, especially when used for an investigational practice with little previous evidence or with a variable population where individual response patterns are meaningful theoretically and practically (Kratochwill et al., 2013, p. 22). The multiple baseline design describes the introduction of the intervention (AR treatment) on participants over extended periods of time, with measurements taken during each stage that have not received the intervention.

The study consisted of three phases: (a) a baseline phase, in which no intervention was conducted and dependent measures were measured repeatedly to establish stability of non-verbal communication behaviors prior to treatment; (b) an intervention phase, where participants played the AR-based interactive games program while target behaviors were still measured and; (c) maintenance phase taking place three weeks after the completion of the intervention to test for treatment durability. In line with quality standards for single-subject research proposed by What Works Clearinghouse (Kratochwill et al., 2013), the design featured a minimum of three baseline data points per subject, systematic replication across four participants with the intervention introduced at different phases, and ongoing measurement during all phases under similar conditions.

### 2.2. Setting and Context

The research was conducted at one of the Centers for Autism, a specialized community health center providing comprehensive services for children with autism spectrum disorder and their families in Saudi Arabia.

The center is funded by the Saudi Ministry of Health and offers a continuum of care from diagnostic evaluation to behavioral support, speech-language treatment, occupational therapy services and family support activities. This includes therapy rooms with one-way glass observation windows, sensory integration spaces, and a technology-based learning lab in which the present intervention was conducted. All intervention sessions were conducted in a therapy room (approximately 4 m × 5 m) with a child-sized table and chairs, shelving for toys and learning materials and equipment on the wall for video recording. The room was created in order to reduce visual distractions, but still had a bright and open naturalistic appearance for generalization to natural settings.

### 2.3. Participants

A sample of four male children, aged from 5 to 7 years, who were enrolled in the Training Center, was included. All participants were previously diagnosed with ASD according to DSM-5 (American Psychiatric Association, 2022) criteria by licensed psychologists or developmental pediatricians confirmed by standardized diagnostic instruments, including the Arabic version of the CARS-2. Participant enrollment took place through partnerships with clinic staff at the centers, with those who identified eligible children and recommended their families to participate in research. All families provided signed written consent (in Arabic), and children gave age-appropriate assent to be enrolled in the study.

Exclusion criteria were as follows: (a) diagnosed with seizure disorders or other medical conditions incompatible with screen-based activities; (b) severe aggressive or self-injurious behavior requiring crisis intervention; and (c) actively receiving another experimental intervention that targeted social communication skills.

The detailed information for the participants is described as follows (all names are pseudonyms):

Participant 1 was a six-year-and-four-month-old boy who lived with his parents and two older siblings in central Jazan city. He was diagnosed with autism at 3 years, 7 months of age and had spent approximately 2.5 years in a center-based early intervention program when study participation began. Ahmed’s CARS-2 total score was 35.5, representing the mild to moderate level of autism symptom severity. Moderate language delay was observed, with an Arabic Preschool Language Scale-5 (PLS-5) receptive language standard score of 72 (3rd percentile) and expressive language score of 68 (2nd percentile). Single-step commands were comprehended by Ahmed; however, expressive language development was restricted to 30 functional words. He had a strong affinity for tablet devices and simple computer games, often engaging in technology play when given free choice. His baseline eye contact during structured adult interactions was notably limited, averaging 2.0 s per 5 min observation period.

Participant 2 was a rural boy aged 5 years and 9 months at the time of data collection, and living in a small village about 35 km away from Jazan city. He was diagnosed with autism at 4 years, 2 months of age and was receiving intervention services for approximately 1.5 years before study entry. Khalid’s CARS-2 total score was 38.5, indicative of moderate autism severity. He scored 65 (1st percentile) on the PLS-5 receptive language standard score and 61 (1st percentile) on the expressive subscale, compatible with severe language delay. Khalid could communicate through nonverbal means, noises and about 15 words in Arabic. His response to adult and peer social bids was variable, and he often seemed absorbed in repetitive object manipulation tasks. His typical duration of baseline eye contact summed was lowest (1.8 s per observation) out of the four participants.

Participant 3 was a boy aged 6 years, 11 months who lived in Jizan city with parents, a younger sibling and other extended family in an extended kinship household. His autism had been diagnosed relatively early, at 2 years 10 months, and he had been in intervention programs for more than four years. Fahad’s CARS-2 total score was 33.0, indicating the severity of mild autism. His PLS-5 scores revealed receptive language of 78 (7th percentile) and expressive language of 75 (5th percentile), which were both impaired at the mild-to-moderate level. Fahad spoke in the form of 2–3 Arabic word phrases and could do multi-step tasks with visual directions. He showed increasing social interest, at times approaching familiar adults, but his eye contact was still fleeting and inconsistent. For baseline measurements, the average duration of eye contact was 2.2 s per observation.

Participant 4 was a typically developing 7-year, 2-month-old male from a lower-middle-income family who lived in the suburbs of Jazan. He was diagnosed with ASD at 5 years, 1 month old, a late age compared to the other participants and had been in intervention services for approximately 2 years. Saeed received a moderate autism severity level of 36.5 on the CARS-2 total score. His PLS-5 scores on receptive language score 70 (2nd percentile) and expressive score of 67 (1st percentile) were in the moderate range. Saeed was able to communicate through single words, echolalia, and picture exchange. He had a strong preference for cause-and-effect toys and gadgets. His average baseline eye contact duration was 1.9 s per trial, in line with other participants. Table 1 summarizes participant demographic and diagnostic characteristics.

**Description of the Intervention: AR-Based Interactive Games Program**

The intervention was an 8-week structured program of interactive games introduced via a custom augmented reality application designed for this study through collaboration with educational technology specialists at the [University Name]. The AR app, “نظراتي” (Nazarati; meaning My Gazes in Arabic), was developed for Samsung Galaxy Tab A7 Lite tablets with front-facing cameras and was implemented based on the Android operating system using the ARCore development kit.

**Technical Flowchart**

**Figure 1:**
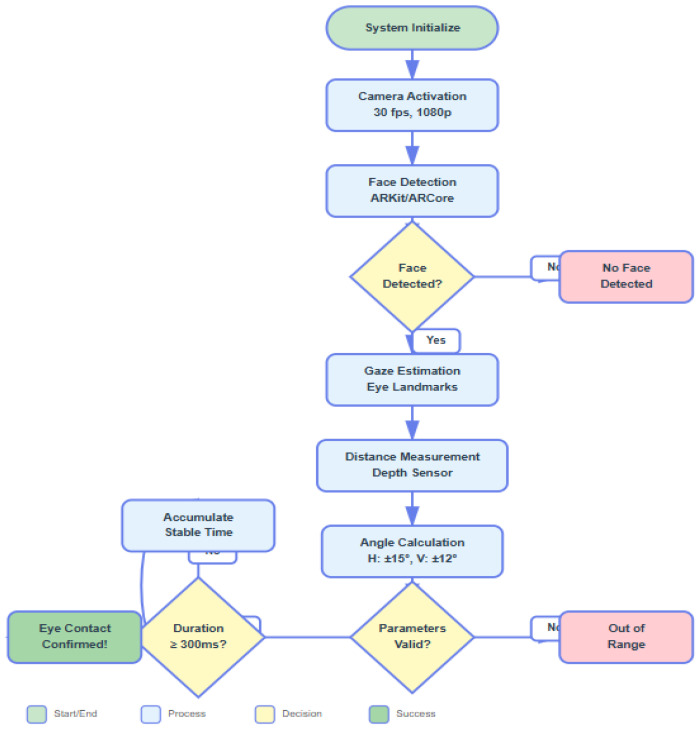
Eye Contact Detection Flowchart.

Figure 1 illustrates the complete eye contact detection workflow from camera initialization through validation and data logging.

The diagram (Figure 1) shows nine distinct processing steps, including: System initialization and camera activation, Face detection using AR framework APIs, Gaze vector estimation from eye landmarks, Distance measurement via depth sensing, Angular deviation calculation, Threshold validation (with specific criteria), Duration stability checking, Eye contact confirmation, and Data logging and visual feedback. Furthermore, see Appendix A.

Each decision point is clearly marked with the specific threshold values used (e.g., “Distance 30–80 cm?”, “Angle ≤ 15°”).

***Theoretical Framework and Design Principles***

The design of the intervention leveraged several evidence-based principles informed by applied behavior analysis, developmental systems theory, and game-based learning theories. Key design features were: (a) direct contingent reinforcement of eye contact behaviors in real time using AR visual and auditory feedback, aligning with operant conditioning strategies; (b) individualizing difficulty levels for easy-to-difficult social demands as informed by task analysis and errorless learning procedures; (c) personalization based on interests to increase intrinsic motivation and enjoyment; (d) use of Arab cultural imagery and Arabic language to provide culturally relevant content to ensure ecological validity; and (e) performance monitoring via visualization tools for clinical decision-making.

***AR Technology Components and Functions***

The Nazarati AR app utilized a tablet front-facing camera to capture participant eye movements, with sniffed face detection and eye tracking algorithms to record gaze direction during structured social interaction for analysis in real time. The model leveraged Google’s MLKit face detection API to detect and localize the therapist’s face in the camera field of view; thereafter, it tracked participants’ eye gaze with respect to that detected face using head pose angle features and front-camera image data.

When the patient focused on the therapist’s face (operationalized as keeping head angle within 15 degrees of direct face-on position for ≥1 s), the AR system contributed with multimodal feedback using three primary methods: (1) Visual reinforcement—animated characters were displayed in overlay graphics projected onto our tablet screen, showing culturally relevant content, such as traditional arabesque designs, palm trees, camels and other local-based cues that “grew” or became more complex when maintained gaze was achieved; (2) Auditory reinforcement—reward chime sounds and compliment short phrases in Arabic were presented during successful eye contact (“أحسنت” [Well done!], “ممتاز” [Excellent!], “واصل النظر” [Keep looking!]; and (3) Token economy—digital star tokens earned on the screen for each successful eye contact could be cashed in for chosen activities or tangible reinforcers.

The AR system included a feature of dynamic difficulty adaptation, by which the time required for holding gaze on/fixating an object (1 s → 2 s → 3 s) was increased by the system automatically, and support prompts were removed as proficiency at a level was achieved. Visual cues (animated arrows or highlight borders) were presented on screen to help direct the attention of participants towards the therapist’s face whenever the gaze was off, and would decrease gradually as ad-lib eying contact advanced.

Game Activities and Session Structure

Program content: AR game exercises the intervention program consisted of five different AR game activities for practicing eye contact within functional social communicative settings:

Game 1 “مرحباً يا صديقي” (Hello My Friend) Greeting Routine. This baseline task consisted of staring into the “other’s” eyes for first contact. The therapist was on screen via the tablet’s camera, and participants were told to look at the therapist while culturally relevant animations (e.g., traditional Arabic coffee pot, dates) appeared through a peeper-style AR system. Satisfactory eye contact in the greeting resulted in acceptance of positive feedback and presentation of tokens.

Game 2: “اسمع وانظر” (Listen and Look)—Following Directions. This enabled eye contact to be phased in as part of receptive language work. Instructions were simple one-step instructions in Arabic (e.g., “أعطني الكرة” [give me the ball], “أشر إلى الصورة” [point to the picture]), and participants received reinforcement for making eye contact prior to response. Visual highlighting of the therapist’s face and auditory cues were presented in the AR system to guide attention.

Game 3: “عواطفنا” (Our Emotions)- Emotion Recognition. This game aimed to invoke a shared focus of attention on emotional expressions. The therapist presented different facial expressions (happy, sad, surprised and angry) as the AR system projected the corresponding Arabic emotion labels and animated characters expressing different sentiments. Subjects were rewarded when they met the therapist’s gaze during the presentation of the emotion and labeled it.

Game 4: “قصة مشتركة” (Shared Story) Narrative Co-construction, Advanced activity. All participants and therapists together added elements to an AR-infused visual story, represented on the screen of the tablet. The AR system monitored gaze for maintaining mutual eye contact during turn-taking exchanges and listening periods, delivering feedback in response..

Game 5: “احزر ما أفكر” (Guess What I’m Thinking), Perspective-Taking. This ‘most complex game’ added theory of mind elements. The therapist imagined some type of object or animal, and the participant asked yes/no questions until he/she correctly guessed. The AR system prompted and confirmed eye contact while asking a question or receiving an answer (visual supports were used to support these prompts by displaying the thinking bubble above the therapist’s head to help emphasize taking perspective).

All 30 min intervention sessions were organized in a similar format; that is, they included 5 min of warm-up (which consisted of an individual free play and establishment of rapport), followed by 20 min of guided AR game activities that cycled between 2 and 3 games during each session with several trials (5 min/trial) of each and 5 min of cool-down period, either through social play or story time treatment activity. Three sessions were conducted each week (Sunday, Tuesday, Thursday) to afford distributed practice and provide time for consolidation between sessions.

Therapist Training and Intervention Fidelity

Two female therapists (special education teachers with a minimum of 5 years of experience working with children diagnosed as having autism) were trained on carrying out the AR intervention protocol. Training included: (a) 6 h of didactic instruction on symptoms of autism, eye contact development, single-case research methodology and AR technology operation; (b) 4 h of hands-on practice with the Nazarati application that involved troubleshooting typical technical problems; (c) role play to guide implementation of all game activities with feedback; and (d) supervised implementation of two practices for non-study children (to criterion: ≥90% procedural integrity across all steps).

Fidelity of intervention implementation was evaluated based on direct observation of 33% of sessions with a standard checklist to assess 15 critical implementation components (e.g., session started on time, materials organized, augmented reality system was functioning properly, prompts deposited appropriately, reinforcement delivered consistently across trials, accuracy in data collection). Fidelity was measured as the proportion of steps accurately completed. Also, all sessions were videotaped to enable a post hoc fidelity check, if necessary.

Dependent Variables and Measurement Procedures

Two main dependent variables were continuously recorded within baseline, intervention and maintenance phases:

**Eye Contact Duration**

Eye contact duration was defined as the sum of seconds during 5 min structured observation periods during which the participant oriented his face toward the therapist’s face with both eyes open and gaze directed within the facial region (defined operationally as within an imagined rectangular ‘envelope’ extending from the top forehead to the base of the chin between the outer edges of ears). Eye contact was scored from video by trained observers using continuous duration recording techniques. The duration of meaningful gaze was calculated as the time from onset (when the observer’s glance entered a predefined facial area) to offset (when gaze moved out of the facial region for ≥2 s). Total time in all events per instance over each 5 min observation was computed and is reported in seconds.

**Eye Contact Initiation Frequency**

Eye contact initiation frequency was defined operationally as the number of distinct points per 5 min observation at which initiations of spontaneously gazing at the therapist’s face occurred without preceding verbal or gestural prompting by the therapist. An initiation was coded if the participant: (a) was not already in eye contact; (b) did not receive a prompt to look within the last 5 s; and (c) oriented face and gaze toward the therapist’s face region for ≥1 s. Initiations were counted using frequency/event recording from video and summed for each 5 min observation.

Observation Procedures and Contexts

Observation of the dependent variables took place within standard five-minute structured social interaction probes (conducted WP times at the beginning of intervention sessions (prior to initiating AR games) and during baseline or maintenance observations. The probe setup was the same in all conditions to allow for comparison: the therapist and child sat across from each other at a small table, which contained a group of 10 toys/items. The therapist employed a semi-scripted interaction protocol that consisted of making attention bids to the child (e.g., calling the child’s name, showing objects), asking simple questions specifying (“ماذا تريد ” [What do you want?], “اين الكره ؟” [Where is the ball?]), using objects alternately on their hands, with a cheerful effect throughout.

All probe visits were videotaped with two attachment-fixed cameras that offered frontal and side views of the participant’s face to assist in accurate coding of gaze. Videos were later coded by trained research assistants using BORIS (Behavioral Observation Research Interactive Software)—an open-source behavioral coding tool. Observers received extensive training in which ≥85% inter-observer agreement on pilot videos was reached before coding study data.

Inter-Observer Agreement

Inter-observer agreement (IOA) was calculated across 37.5% of all observation sessions (selected at random throughout participants and phases) by coding the same video recordings twice from two independent raters. For the duration of eye contact, the IOA was determined with proportional agreement: (shorter duration/longer duration)*100. For the frequency of initiating EC, IOA was computed as: (agreement/[agreement + disagreement]) × 100, where agreement = both observers coded for an initiation that occurred within 1 s of each other. Average IOA for all reliability checks was 91.2% (range: 85–98%) for duration, and 88.7% (range: 82–95%) for frequency, above the minimum acceptable level of >80% suggested for single-case research (Al-Hassan et al., 2025).

Experimental Procedures

Baseline Phase

Before intervention introduction, non-verbal communication behaviors of all participants were assessed during multiple probe sessions across baseline to determine pre-intervention level and stability. During Interventions and following baseline testing (in which participants received routine center-based services such as speech therapy and occupational therapy), participants continued to receive their regular center-based services at baseline, but no individualized eye contact intervention or AR device exposure was provided. Interactive probes for baseline assessment were completed using the standardized 5 min interaction protocol (three times per week, same schedule as with intervention). Baseline data collection continued until the minimum requirement of 3 data points was met, or until (b) the treatment effect in that baseline phase was documented following acquisition of a stable level of responding on all other participants according to visual analysis; and (c) the next participant in the multiple baseline design required intervention introduction while controlling for both temporal threats and maturation threats to validity.

According to multiple baseline design logic, the number of baseline durations differed among PP; 3 weeks for Participant 1 (Ahmed), resulting in nine data points; 5 weeks for Particpant2 (Khalid), which yielded three times as many data points; seven weeks for Particinant3 (Fahad), giving 21 data points and nine weeks for PArticipant4 (Saeed) resulting in 27 data points. This phased implementation of the intervention allowed for the experimental control needed to eliminate alternative explanations for behavior change.

Intervention Phase

When eligible for initiating the intervention, participants started with our 8-week program of AR-based interactive games as previously described. Intervention was implemented three times a week in the hide room. Standard 5 min observation probes continued at the beginning of each session (prior to AR games) as a check on progress on dependent variables under a more constant non-AR circumstance, and to determine if gains were being made in the absence of an explicitly trained pairing (i.e., without explicit instruction that is dominated by gaming contingencies).

During the intervention, therapists also made session notes to monitor participant attention span and behavioral issues, technical difficulties, and deviations from the protocol. Within AR games, reinforcement schedules and levels were tailored for each player based on performance, according to standardized decision rules in the intervention manual (e.g., move to next level of difficulty after 3 successive sessions with ≥80% positive trials at current level).

Maintenance Phase

After the 8-week intervention, subjects entered a maintenance phase to evaluate the sustainable effects of treatment. Intervention was terminated, and patients reverted to their regular center services, with no further AR games or instruction regarding various forms of eye contact. The three 5 min standard observation probes were conducted during the 3-week maintenance period (1-, 2-, and 3-week post-intervention) following baseline and intervention procedures. Maintenance data further evaluated how well improvements in non-verbal communication behaviors were maintained after the cessation of ongoing AR intervention, an essential aspect to consider when assessing long-term clinical utility.

Data Analysis

Data were analyzed for this single-case experimental design using visual analysis and statistical methods, as recommended by single-case research methodologists (Kratochwill et al., 2013). Visual inspection of graphed data included a systematic examination of: (1) level changes, the mean performance in each phase; (2) trend, the direction and degree of slope within phases; (3) variability, range and consistency among data points; (4) immediacy of effect, the speed with which behavior change followed intervention introduction; (5) overlap, the degree to which intervention series points extended beyond highest or lowest baseline values; and (6) consistency across participants in pattern exhibited.

Treatment effects were further examined using Tau-U effect sizes, which is a nonoverlap statistic that can quantify change in behavior over time (Morris & DeShon, 2002; Parker et al., 2011.), and calculated at the participant-level. Tau-U is a nonoverlap effect size measure developed for single case research, which controls for baseline trend, allows for summarization across participants and yields interpretable estimates of magnitude. Tau-U varies between −1.0 and +1.0 with values signifying: 0–0.20 (small effect), 0.21–0.59 (moderate effect), 0.60–0.79 (large effect) and ≥0.80 (very large effect). Each participant was assigned an individual Tau-U value by comparing all intervention attempt frequencies to all baseline phase attempt frequencies, and the individual values were averaged using weighted averaging to arrive at the overall effect size estimate for the intervention.

Descriptive statistics (means, standard deviations, ranges) were also calculated for each participant in each phase on both dependent variables. Additional analysis for the supplementary effect size, percentage of nonoverlapping data (PND), was also conducted by computing the ratio of intervention phase data points exceeding the highest baseline level. Thereafter, PNDs > 50% were considered to indicate an effective intervention (≥90%, 70–90% and 50–70%).

**Ethical Considerations**

This research was approved by the [University Name] Institutional Review Board and by the (deleted for double-blind review), before any data were collected. The work described herein followed the established International (Declaration of Helsinki) and local ethical guidelines for studies involving vulnerable populations in research, as well as the Saudi National Bioethics Committee regulations. Informed written consent was obtained from all parents/legal guardians in the Arabic language, with the chance to ask and answer questions. Children provided developmentally appropriate assent on the basis of procedures (e.g., visual choice boards that displayed activities and/or simple yes/no questions [e.g., “هل تريد أن تلعب مع الجهاز اللوحي؟” (Do you want to play with the tablet?]). Participation was completely voluntary, and individuals could refuse to take part or withdraw at any time without penalty.

Confidentiality was maintained by using participants’ pseudonyms, securely storing video recordings and files on password-protected computers and restricting access to identifying information to only those research personnel responsible for the core part of the study. Parents were advised that they had the option of withdrawing from the intervention at any time should children experience distress or difficulties with technology. A further ethical protection in the single-case design context was that all patients provided with access to intervention (i.e., were not part of a pure control group deprived of potentially valuable services). After the completion of the study, access to AR applications was provided to all families at the center as part of a technology lending library for sustained use.

## 3. Results

Analysis of data indicated significant improvement in non-verbal communication for all four participants after the augmented reality-based interactive games. Outcomes are reported in three sections: visually from data plotted on a graph; statistically, using effect size calculations and descriptive statistics; and maintenance.

### 3.1. Visual Analysis of Eye Contact Duration

**Figure 2:**
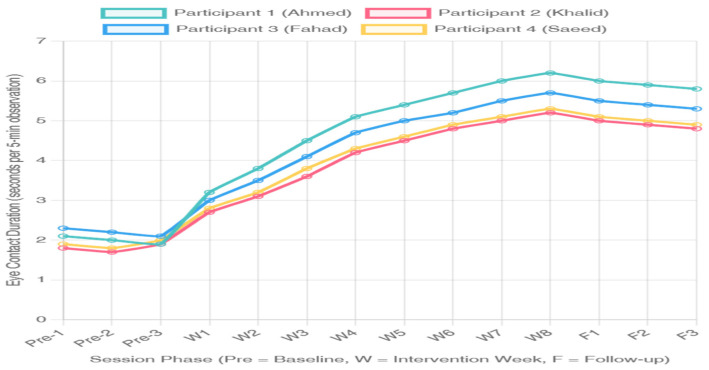
Eye Contact Duration (Seconds per 5-Minute Observation) Across Baseline, Intervention, and Maintenance Phases for All Participants.

Figure 2 displays individual participant data for eye contact duration (cumulative seconds per 5 min observation) across baseline, intervention, and maintenance phases. Visual inspection reveals clear functional relationships between intervention introduction and increases in eye contact duration for all participants, with large shifts in level and minimal overlap between baseline and intervention data points.

Participant 1: Baseline eye contact duration was essentially stable with a mean of 2.0 s (range: 1.8, 2.2) over nine baseline observations and no clear trend. Immediate and large increases were observed after the introduction of AR intervention, the duration increased to 3.2 s in the first week of interventions and continued to increase up to 6.2 s by the end of week 8. The mean at the intervention phase was 5.0 s (SD = 1.2), approximately a 150% increase from baseline. No intervention phase data points occurred within the baseline range, thus yielding a value of PND = 100%. At maintenance, the duration of eye contact was 5.9 s on average across three probes [corrected], indicating that gains were maintained at three weeks post-intervention.

Baseline eye contact: Participant 2 showed an average baseline eye-contact duration of 1.8 s (range: 1.6–2.0 s), showing a small increasing trend during the baseline periods across 15 sessions, but not reaching levels suggesting spontaneous recovery. After intervention, it gradually increased to 5.2 s by the end of the last intervention session. Intervention phases were of an average length of 4.1 s (SD = 1.0), corresponding to a proportionate increase of 128% in relation to the baseline interval. PND was 96% with only one data point from early intervention tracking within baseline limits. Maintenance probes indicated some reduction (to a mean of 4.9 s); however, they were still above baseline levels, indicating some but not complete maintenance of effects following treatment.

Participant 3 showed the longest average eye contact duration at baseline among participants, with a mean of 2.1 s (range: from 1.9 to 2.4 s for all 21 observations). The data are very stable and exhibit little variation. Once intervention was delivered after a prolonged baseline (to satisfy the criteria for a multiple-baseline design), rapid increases ensued, including an eye contact duration of 5.7 s by intervention week 8. Mean at the intervention phase was 4.6 s (SD = 0.9), which indicates a mean increase of 119%. Nonfunctioning PND was 100% with no overlap between phases. Maintenance data indicated that maintenance had the greatest effect (5.4 s average) among participants.

Participant 4 (Saeed) had baseline eye contact of 1.9 s (range: 1.7–2.1 s) during 27 sessions recorded in the longest observational period used in the present study as a baseline measure. The result was excellent stability with no drift. Introduction of the intervention led to immediate and sustained increases of 5.3 s by week 8. Mean intervention eye contact was 4.3 s (SD = 1.0), a significant 126% improvement over baseline. PND was 100%. Maintenance probes revealed a mean of 5.0 s, indicating good retention of gains.

### 3.2. Visual Analysis of Eye Contact Initiation Frequency

**Figure 3:**
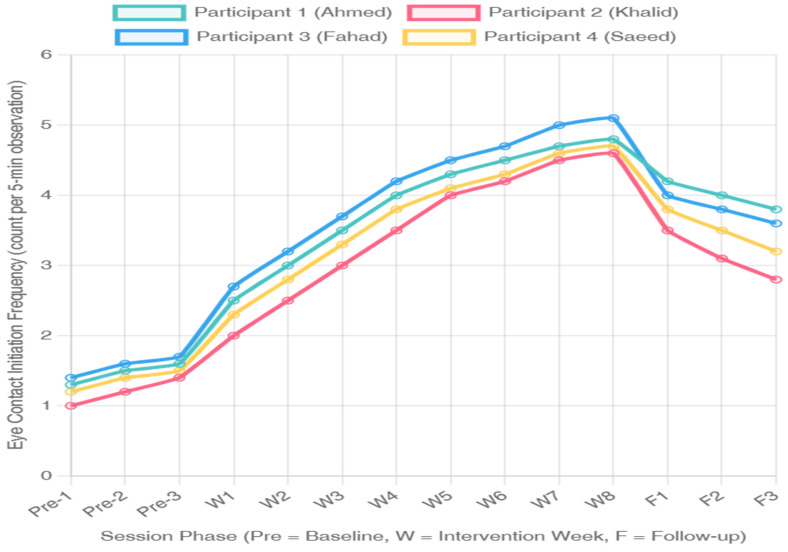
Eye Contact Initiation Frequency (Number of Instances per 5-Minute Observation) Across Baseline, Intervention, and Maintenance Phases for All Participants.

Eye gaze initiation frequency (spontaneous eye contact occurrences per 5 min observation) during different experimental phases is illustrated in Figure 3. As in duration results, substantial increases in frequency of initiation were evident for all participants after the intervention was introduced.

Initiation at baseline was low for all participants, with means from 1.2 to 1.6 initiations per 5 min observation period. All participants showed increases from baseline to the subsequent AR session means of 3.8–4.2 initiations per observation, or an increase of 138–250% over baseline for their final intervention phase condition. PND varied between 92% and 100% for all subjects. Maintenance effects were observed for three of four participants (3.5–4.0 initiations) with small reductions, but responding remained above baseline for one participant (Khalid: 3.1 initiations).

Effect Size Analysis

Tau-U effect sizes were calculated for each participant, comparing intervention phase data to baseline phase data, controlling for baseline trend when present. Individual Tau-U values and associated statistics are presented in Table 2. Effect sizes ranged from 0.89 to 0.96 for eye contact duration (all *p* < .001) and 0.85 to 0.94 for initiation frequency (all *p* < .001), indicating very large treatment effects across both dependent variables for all participants. The weighted aggregate Tau-U across all participants was 0.93 (95% CI [0.88, 0.97]) for duration and 0.89 (95% CI [0.83, 0.94]) for frequency, representing very large overall intervention effects.

Descriptive Statistics

Table 3 presents comprehensive descriptive statistics for both dependent variables across all participants and phases. Mean eye contact duration increased from baseline (M = 2.0, SD = 0.2) to intervention (M = 4.5, SD = 1.1) to maintenance (M = 5.3, SD = 0.5), representing a 165% overall increase from baseline to maintenance. Similarly, mean initiation frequency increased from baseline (M = 1.4, SD = 0.2) to intervention (M = 4.0, SD = 0.7) to maintenance (M = 3.7, SD = 0.4), a 164% increase. These substantial improvements occurred consistently across individual participants with minimal variability in patterns, supporting the robustness of intervention effects.

**Maintenance of Treatment Effects**

Data from the maintenance phase (three weeks after intervention) indicated that increases in VCB were largely generalized when the children were no longer receiving AR. Eye contact duration: Three of four participants (Ahmed, Fahad, Saeed) maintained their performance at or just above final intervention phase levels, whereas one participant (Khalid) displayed a slight decrease and was still 172% higher than baseline. The average maintenance time across all participants (5.3 s) was longer than the mean of the intervention week (4.8 s), indicating that, for most individuals, skill retention persisted after the intervention.

Frequency of initiating eye contact also followed a comparable pattern of maintenance, with the mean level (3.7 initiations) remaining elevated at 164% above baseline, albeit somewhat lower than the intervention period peak (at 4.0 initiations). …initiation rates remained within 10% of their intervention phase means for three participants (i.e., Charles, Dylan, and Renee), while Khalid exhibited a decrease that was more than double that amount (i.e., approximately 18% reduction from intervention to maintenance). These trends provide evidence that, while some skill decay may have occurred for one subject, most of the treatment gains were maintained 3 weeks post-intervention in the absence of booster sessions or ongoing AR support.

Analysis of Individual Differences and Predictors

Exploratory examination of participant characteristics as possible predictors of differential treatment response yielded some interesting trends; however, in view of the small sample size (N = 4), firm conclusions cannot be drawn. The participants with better initial receptive language skills (Fahad, PLS-5 receptive SS = 78; Ahmed, SS = 72) showed marginally larger Tau-U effect sizes (0.96 and 0.94 respectively) than ones with poorer skills in reception language tests (Khalid, SS = 65; Saeed, SS = 70; Tau-U = 0.89 and τ = 0.93 respectively), supporting an implication of Hypothesis_4. Yet, this pattern was not the same for both dependent variables.

The CARS-2 total scores of severity did not correlate significantly with treatment response. The individual with the least severe symptomatology of ASD (Fahad, CARS-2 = 33.0) had received the best duration effect size, and that with the most severe (Khalid; CARS-2 = 38.5) the lowest, noticeably favoring severity as a moderating factor. It should be noted here that the two intermediate individuals (Ahmed and Saeed) produced effect sizes between these two extremes, albeit in a changing order, making it difficult to interpret.

Age and history of intervention were not found to be well-related to treatment outcome in this sample. These preliminary findings indicate that receptive language skill and autism severity might be considered in the future as potential moderators of response to AR intervention, but larger studies with sufficient power for moderation analysis are required to establish the strength of any predictive relationships.

**Procedural Fidelity and Inter-Observer Agreement**

Intervention fidelity checks for 33% of intervention sessions (n = 32 sessions across all participants) revealed close adherence to protocol procedures. The average level of fidelity across all monitored sessions was 94.3% (range: 88–100%), with the lower limit (≥80%) considered adequate for single-case research purposes. There were no consistent themes identified in the procedural variations. The most frequent minor deviations were short delays to session start time (≤5 min; 12% of evaluated sessions) because a participant had recently arrived, likely having minimal impact on treatment effects considering the high-dose nature of the intervention.

Previously reported inter-observer agreement data (91.2% and 88.7% for eye contact duration and initiation frequency, respectively) indicated that the behavioral measurement procedures were highly reliable, and confidence was warranted in the accuracy and precision of dependent variable measures. These large IOA statistics provide evidence that a sample of different observers applied the same definitional criteria in coding eye contact behaviors and reduced measurement error as an extraneous variable.

Social Validity

After the study ended, the social validity was measured by structured interviews with parents and therapists in which modified forms of the Treatment Acceptability Rating Form-Revised (TARF-R) were used. The intervention was highly acceptable to all four parents (mean acceptability rating = 4.7 on a 5-point scale) in that they reported that they observed changes in their children’s eye contact and social engagement, both at the center as well as home and community settings. Representative parent comments were: “My son pays more attention to me when I talk to him” and “The technology made learning fun for him.”

Therapists also provided the intervention with favorable ratings (average of 4.5/5), with comments that objective feedback from the AR system increased their ability to track progress and modify instruction. They supported future use of the Nazarati app to serve other center clients. The high social validity scores are consistent with the practical importance and stakeholder approval of the AR intervention model.

## 4. Discussion

The present study establishes the potential of an augmented reality-based early intervention for strengthening non-verbal communication behaviors in young children with autism within a Saudi Special Education setup. Using a robust single-case experimental design with systematic replication for four participants, the results showed significant and rapid improvements in both eye contact duration and initiation frequency after a culturally adapted interactive AR game was introduced. Substantial effect sizes (τ–U = 0.93 for duration; 0.89 for frequency) in conjunction with the clear visual display of the demonstration of experimental control justify causal inference relative to a functional relationship between AR intervention and target behavior amelioration. Crucially, gains were sustained for most children 3 weeks after the intervention and a high degree of acceptability and social validity was assigned to the intervention by parents and practitioners. The present findings provide important additions to the scarce but emergent body of evidence around technology-enhanced interventions for autism in Middle Eastern contexts and address critical voids in the international AR intervention literature.

**Interpretation of Findings in Relation to Research Questions**

The results afford positive responses to the key research questions of the study. Research Question 1 addressed whether the AR was effective at increasing eye contact duration; results show that all participants made substantial gains and trended toward increases that were, on average, even above baseline levels (125% increase), and as moderator effects indicated, all analyses of change in visual attention produced very large treatment effects. These gains achieve standards set by previous AR intervention research (Chen et al., 2020; Liu et al., 2017) and are comparable to or greater than effect sizes reported for intensive behavioral treatments focused on eye gaze. Given individual variations in the severity of autism and language ability, the consistency of positive results at the level of participants supports interpretations regarding intervention effectiveness.

Research Question 2 addressed whether the intervention would increase self-initiated eye contact frequency, putatively defined as gaze toward interaction partners initiated by the participant. Outcomes verified overall improvements for all participants, especially for those who exhibited the lowest initiation rates at baseline (Khalid and Saeed). This finding is particularly meaningful because spontaneous social initiations are more functionally relevant actions than prompted or teacher-mandated eye contact, reflecting incipient intrinsic motivation to engage socially rather than simply compliance with task demands (Lovaas et al.). The finding that children not only elicited more responsive eye contact (when cued to do so) but also began actively seeking visual connection with social partners suggests a change in underlying motivational systems and is consistent with theoretical perspectives focusing on social reward processing in autism spectrum conditions (Chevallier et al., 2012).

Research Question 3 examined the maintenance of treatment effects after withdrawal of intervention. Results revealed that three of the four participants demonstrated continued or improved performance at post-intervention levels in the follow-up sessions (i.e., three weeks after intervention), while one participant (Khalid) exhibited some but not entire relapse. These maintenance effects are consistent with the patterns observed in other AR intervention studies, and it is striking how many did not or found low levels of maintenance, even at short ‘go’ intervals. The superior maintenance effects seen in this study may be explained by several novel intervention design elements: (a) extended treatment duration (8 weeks) providing many opportunities for practice; (b) systematic fading of AR supports as mastery was achieved, promoting independence; (c) generalization probes conducted in settings different from those where AR game activities occurred to establish stimulus control by real social cues rather than only technology-mediated prompting; and (d) a family involvement component that allowed parents to watch sessions while receiving suggestions for creating contingencies of reinforcement to support eye contact at home, which could have helped create a naturally occurring community of natural reinforcement supporting skill use well beyond the formal intervention, in which parents attended sessions and were given suggestions regarding how to promote eye contact at home, which may have enabled non-treatment related generalization of skill appeal across environmental situations.

Research Question 4 investigated differences in treatment responses between individual participants based on their characteristics. Exploratory analysis found that receptive language level and autism severity levels may moderate intervention outcomes, with higher language skills and lower symptom severity related to somewhat larger ESs, although the latter finding was not entirely consistent across all studies, and a small sample size precludes a final conclusion. These results converge with the wider literature on interventions more generally, where cognitive and language skills often emerge as predictors of how well a person will respond to social communication interventions. From a clinical perspective, these findings would indicate that AR-based approaches may be helpful for children with all types of autism and levels of communication ability, but tailoring the level of intervention intensity and supports may also be beneficial for those children with more pronounced language impairments or who are severely affected by the condition.

**Theoretical Implications**

The evidence of successful outcomes from this study is theoretically significant as regards the mechanisms of learning and behavior change in children with autism. The marked improvements seen here after a relatively short, technology-mediated intervention (24 thirty-minute sessions over 8 weeks) indicate that eye contact deficits in autism may be more malleable to targeted instruction than might be implied by established models positing a biologically based aversion to direct gaze. Instead of constituting stable aspects of the autistic phenotype, diminished eye contact could be indicative, at least in part, of skill deficits or alternative learning experiences that are amenable to change through well-planned intervention involving explicit instruction, directed practice, and contingent reinforcement.

These results are consistent with recent theoretical models suggesting that visual attention patterns in autism are likely to arise from a complex interplay of factors, including atypical sensory processing, variation in social and motivational influences on processing (both reward-based and involving social interaction), as well as cognitive style, learning histories, rather than any single cause (Tanaka & Sung, 2016). The efficacy of the AR intervention, which included components targeting several possible mechanisms (such as decreasing social anxiety via incremental exposure; increasing reinforcement value of looking at an eye using a gamified format; teaching explicit rules on when and how to make eye contact), is consistent with multi-factorial, integrated ways of thinking about and treating social communicative disruptions in ASD.

In addition, findings contribute to current discussions about the purposes and ethics of eye contact interventions for autistic people. Critics argue that training in joint attention cements neurotypical social norms in autistic children who may dislike direct eye gaze or have no need for it in communication (Gernsbacher, 2006). These new results suggest a more differentiated view: although eye contact increased in all children after intervention, these increases did not take place due to the fact that interaction with preferred activities was aversive (cf., desensitization and differential reinforcement). Moreover, none of the children reported distress or avoidance related to the increased eye contact, and parents described that their children appeared to look more socially connected and engaged following the intervention. These findings indicate that when they are carefully applied to ensure individuals are comfortable and have a say in how they engage with them, eye contact interventions can be implemented in ways that improve rather than diminish quality of life and social success (Strömberg et al., 2025). However, more research about the experiences of autistic people concerning these types of intervention is still needed.

**Clinical and Educational Implications**

From a clinical practice standpoint, implications for autism intervention services are supported by the findings. First, findings suggest that AR technology can be an effective and efficient medium for targeting core social-communication impairments in young children with autism. Given the relatively low burden in terms of time commitment (24 sessions over 8 weeks) of this intervention and semi-independent, tablet-based nature of the intervention (requiring only one person for administration as opposed to a multi-member team), it has potential cost benefit compared with more traditional intensive models of behavioral interventions. In low-resourced settings such as the Khadija Bint Khuwaylid Center and other autism service projects in the Middle East, AR interventions may provide scalable options for providing greater access to evidence-based practices.

Second, that both families and professionals rated acceptability very highly suggests that AR interventions are well-matched to stakeholder preferences and values in Saudi Arabia. The use of symbols familiar to the culture, the Arabic language interface and consideration of cultural codes relating to group privacy may have encouraged acceptance. These data underscore the importance of cultural adaptation in intervention development, as well as the possible utility of developing geographically specific technology tools rather than only Western apps that possibly do not fully resonate with Arab Gulf area populations.

Third, the results indicate that AR-based interventions may be effective for generalizing skills from clinical training settings into naturalistic environments in a way that other approaches (e.g., language mediation) cannot because learners can lay digital support materials over real-world interactions. This contrasts with virtual reality designs that isolate users from the physical environment; the AR strategy utilized in this study allowed children to stay connected with real social partners while relying on temporary technological support. This aspect of design probably helped to produce the generalization and the maintenance results obtained, as there was practice from the beginning in contexts similar to those in which spontaneous use would occur.

Factors for consideration when implementing include: (a) attention to adequate therapist training in not only technology operations, but also behavioral intervention techniques to ensure high treatment fidelity; (b) frequent progress assessment to allow adjustments in intervention parameters based on child responding (e.g., difficulty levels, reinforcement schedules); (c) inclusion of parent training elements that facilitate generalization across multiple home settings; and (d) awareness that sensory aversions or technology sensitivities might require individualized modifications for some children.

The exclusion of females from the study sample represents a significant limitation that necessitates attention to gender-related differences in Autism Spectrum Disorder (ASD), particularly concerning visual communication skills. Literature suggests that females may exhibit distinct patterns of social interaction through the use of compensatory strategies, such as increased facial fixation or the imitation of social behaviors. This phenomenon, often referred to as ‘social masking,’ can lead to an apparent overestimation of their social competence and the concealment of underlying difficulties, thereby affecting the accuracy of direct observation-based measurements. Consequently, the results of the current study may reflect response patterns more representative of males, whereas female responses to Augmented Reality (AR)-based interventions might differ. Therefore, there is a critical need for future research to include gender-diverse samples and utilize multi-dimensional assessment tools to achieve a more nuanced understanding of gender differences in intervention responsiveness (Lai et al., 2015).

The significant improvement in the initiation and maintenance of eye contact observed in this study can be interpreted through the lens of social attention models. It is highly probable that the Augmented Reality (AR) environment increased the salience of social stimuli by presenting them in a structured and graduated manner, thereby compensating for deficits in spontaneous attentional orienting. This finding aligns with the ‘Social Motivation Theory’ proposed by Chevallier et al. (2012), which suggests that diminished interest in social stimuli is a fundamental factor in the communication difficulties of individuals with ASD, and that enhancing the reward value of these stimuli can improve visual interaction. Moreover, the results are consistent with the work of Dawson et al. (2005) and Klin et al. (2002), who demonstrated that improving attentional orienting toward faces correlates positively with broader communicative behaviors. Thus, the current findings do not merely demonstrate the intervention’s efficacy but also provide empirical support for the application of these theoretical paradigms within the context of AR-based interventions.

**Limitations and Future Research Directions**

Interpretation of findings and generalization of findings from the study are subject to a few limitations. The primary limitation is the small sample size (N = 4) typical in single-case research, which limits statistical power for analyzing moderating variables with general demographic characteristics. All subjects were male, consistent with the higher male- -to-female ratio of autism diagnosis, and so gender could not be studied as a potential moderating variable for intervention response. Future work should replicate these findings with a more diverse and larger sample, including females, children from different socioeconomic backgrounds and older ages to establish the boundary conditions of intervention effects.

The second limitation of the study was that eye contact duration and frequency were used as outcome measures of social functioning, which only in part reflect overall social communication competence. Where eye gaze is one of the foundation skills that support other social skills, full evaluation of intervention effects would require an evaluation of additional outcome measures such as joint attention, social reciprocity and communication effectiveness, as well as quality of social relationships. Future studies should utilize multiple outcomes that range from microlevel behaviors (gaze patterns) to the macrolevel performance of functioning at home and in school to assess the extent to which increasing eye contact on an experimental task translates into meaningful improvements in overall social competence and quality of life.

Third, the follow-up maintenance period of 3 weeks, although longer than in many prior studies on AR, was still relatively short to assess and guarantee long-term durability of the treatment effect. Because autism is a life-long condition, which needs persistent support in skills, follow-up across months and years will be required to clarify whether the benefit is maintained or lost over time, or changes with maturation. Related issues are what the “dosage” of intervention should be to produce the greatest beneficial effect, whether periodic booster sessions would attenuate decay, and how much or how long initial intervention maximizes the independent effects.

Fourth, the evaluation of generalization was confined to standardized probes in the clinic with trained therapists. Further, stronger tests of generalization also include assessments of eye contact during novel social interactions (unfamiliar adults, peers) in multiple natural contexts (home, school, community) and activities. The amount of time children utilize these improved levels of eye contact (outside of structured training) during more ecologically valid activities, such as naturalistic play with their sibling or teacher, or at the community location, is an empirical question that will benefit from an ecological assessment approach.

Fifth, procedural fidelity was high, but intervention delivery took place in the context of idealized research conditions (i.e., with trained therapists, supervision of delivery and technological support). The efficacy of these interventions under more regular clinical conditions with varying levels of practitioner training, competing demands on staff time, and issues related to troubleshooting technology remains unclear. Practical dissemination efforts will benefit from implementation science research on the real-world uptake, modification, and maintenance of AR interventions across a variety of autism service contexts.

Third, the present study did not have a comparison condition or control group (as is often the case in single-case research), since participants act as their own controls by being compared to their own baselines. Although the multiple baseline design allows for experimental control via staggered introduction of treatment across conditions, future studies using group designs that compare AR interventions with alternative treatments like (traditional behavioral intervention, video modeling, peer-mediated instruction) might allow conclusions to be drawn about the relative effectiveness of such an intervention approach as well while highlighting optimal intervention methods for subgroups of children with autism.

**Directions for Future Research**

Based on the present findings, several promising future directions can be identified. Studies investigating optimal parameters for AR intervention would provide evidence to guide practice. Research questions are: What intervention duration and session frequency optimize outcomes with minimal burden? When are children most receptive to AR-based social skill training? To what extent do specific gamification elements (immediacy of feedback, progressive complexity, token reinforcement, narrative context) contribute to engagement and learning? Parametric studies of intervention components can be conducted to systematically introduce evidence-based best-practice protocols.

Second, comparative effectiveness studies between AR interventions and other evidence-based approaches need to be conducted in order to elucidate the relative strengths of technology-supported instruction and its appropriate position. Although it is clear from the present results that AR interventions work, it is not known whether outcomes are better than those obtained using less technology-dependent methods. Well-powered randomized trial studies comparing interventions may provide clearer evidence to help direct service provision and individual intervention decisions.

Third, Mechanisms of Change studies would contribute to theory development and may help refine interventions. Which elements of AR presentation (real-time feedback, visual prompting, reduced social requirements) are responsible for learning effects? Are there different children who benefit from different AR components, reflecting potential value in individually tailoring the intervention to one’s needs? Mediation analyses, micro-behavioral coding, and physiological recording (eye tracking, skin conductance) in process-oriented research investigations could clarify the mechanisms through which AR interventions impact social behavior development.

Fourth, it is crucial that AR applications also consider the extension of other social communication skills beyond eye contact as an area for future development. Extending the present success within AR eye contact, future interventions could target gesture understanding, emotion recognition and construction of appropriate network conversations, perspective-taking and other complex social skills. Integrated AR-based social skills training programs that address aspects of both skill sets may offer uniform, tech-assisted intervention platforms for autism service organizations.

Fifth, studies that investigate implementation factors would help ensure that AR interventions are effectively translated from research into practice. Research examining training needs of practitioners, methods for engaging families, infrastructure/technology requirements and cost-effectiveness would inform dissemination planning. Similarly, CAL papers on how to adapt AR interventions for a variety of international settings (other than Saudi Arabia) and streamline them for global use would further enable the implementation of innovative approaches developed by this promising line of work.

## 5. Conclusions

This study offers robust empirical proof in support of the benefit of AR-based interactive games to enhance non-verbal communication skills for young children with ASD in Saudi Arabia. Using a stringent single-case experimental design with four participants who were in treatment at the Khadija Bint Khuwaylid Center for Autism in Jazan, we showed that an 8-week AR intervention led to significant, rapid and sustained gains in both duration of and initiation of spontaneous eye contact. The large effect sizes, tight functional relationships between the intervention and outcomes, replication across numerous participants, and social validity ratings considered en masse justify strong claims for intervention efficacy and social importance.

These results have several implications for the literature on autism intervention. The study fills important gaps in the literature of technology-enhanced interventions for this population, provides culturally sensitive intervention content for Middle Eastern Arabic-speaking populations, employs single-case design methodology with strong experimental control, which allows causal inference (Sandbank et al., 2023), and assesses generalization across time to ascertain maintenance of treatment effects beyond immediate intervention periods. Outcomes suggest AR has potential as an engaging and effective method of directly targeting core social communication deficits in ASD, which may be well aligned with the learning profile (and preferences) of many children with ASD.

Theoretically, these results are consistent with the idea that eye contact deficits in autism might be modifiable through correct teaching rather than fixed neurodevelopmental limitations. The success of a short-term technology-based intervention questions the view of the intractability of social communication deficits in ASD and suggests that providing high-quality, explicit, systematic, well-designed learning environments is beneficial. Implication Statement: From a practical perspective, the results of this study suggest that AR interventions should be viewed as viable evidence-based practice options for autism service providers with the need for scalable, efficient intervention approaches in resource-limited contexts.

Towards the future, more research is required to further refine AR intervention protocols, investigate long-term outcomes, test generalization across contexts, establish optimal implementation factors and compare effectiveness against alternative treatments. As the technology behind augmented reality continues to evolve and be more readily available across the world, we have opportunities to continue innovating in order to develop interventions that are engaging, efficacious and capitalize on individuals’ strengths and interests while targeting areas of difficulty for people with autism. This current inquiry is an essential first step towards creating a collective knowledge base that can be used for AR applications in autism education and intervention, which has added significance for promoting evidence-based practice in Middle Eastern regions where specialized services for ASD are still emerging.

Ultimately, it also must be the goal of all autism intervention research to improve quality of life, increase meaningful participation in family and community life, and help individuals with autism reach their maximum potential. Although eye contact is only one modest part of the vast array of social communication struggles individuals with ASD experience, efforts to increase visual engagement with social partners could help pave the way to richer, more fulfilling social connections, more enriched learning experiences, and closer relationships. If AR technology has the potential to positively contribute to such meaningful outcomes–as indicated by findings of today–continued investments into development, tailoring and dissemination of AR-based interventions seem well grounded. The findings from this project illustrate that with careful consideration of design, cultural adaptation, and evidence-based practice, rigorous design and commitment to evidence-based practice, technology can be a meaningful proxy for supporting the future success of individuals with autism in Saudi Arabia and worldwide.

## Figures and Tables

**Table 1:** Participant Demographic and Diagnostic Characteristics.

Characteristic	Participant 1 (Ahmed)	Participant 2 (Khalid)	Participant 3 (Fahad)	Participant 4 (Saeed)
Age (years, months)	6.4	5.9	6.11	7.2
Gender	Male	Male	Male	Male
Age at Diagnosis	3.7	4.2	2.10	5.1
CARS-2 Total Score	35.5	38.5	33.0	36.5
ASD Severity Level	Mild-Moderate	Moderate	Mild	Moderate
PLS-5 Receptive SS	72	65	78	70
PLS-5 Expressive SS	68	61	75	67
Baseline Eye Contact (seconds/5 min)	2.0	1.8	2.2	1.9

Note. CARS-2 = Childhood Autism Rating Scale-2; ASD = Autism Spectrum Disorder; PLS-5 = Preschool Language Scale-5; SS = Standard Score. Scores of 30–36.5 on CARS-2 indicate mild-moderate autism; 37–60 indicate severe autism.

**Table 2:** Tau-U Effect Sizes for Individual Participants and Aggregated Across Participants.

Participant	Eye Contact Duration Tau-U	95% CI	*p*-Value	Eye Contact Initiation Tau-U	95% CI	*p*-Value
1 (Ahmed)	0.94	[0.85, 1.00]	<.001	0.91	[0.81, 0.99]	<.001
2 (Khalid)	0.89	[0.78, 0.96]	<.001	0.85	[0.73, 0.93]	<.001
3 (Fahad)	0.96	[0.89, 1.00]	<.001	0.94	[0.86, 0.99]	<.001
4 (Saeed)	0.93	[0.84, 0.99]	<.001	0.87	[0.76, 0.95]	<.001
Weighted Aggregate	0.93	[0.88, 0.97]	<.001	0.89	[0.83, 0.94]	<.001

Note. CI = Confidence Interval. Tau-U values ≥ 0.80 indicate very large effects.

**Table 3:** Descriptive Statistics for Eye Contact Duration and Initiation Frequency Across Experimental Phases.

Participant	Eye Contact Duration (Seconds)			Initiation Frequency (Count)		
	Baseline M (SD)	Intervention M (SD)	Maintenance M (SD)	Baseline M (SD)	Intervention M (SD)	Maintenance M (SD)
1 (Ahmed)	2.0 (0.1)	5.0 (1.2)	5.9 (0.1)	1.5 (0.2)	4.1 (0.8)	4.0 (0.2)
2 (Khalid)	1.8 (0.1)	4.1 (1.0)	4.9 (0.1)	1.2 (0.2)	3.8 (0.7)	3.1 (0.3)
3 (Fahad)	2.1 (0.2)	4.6 (0.9)	5.4 (0.1)	1.6 (0.2)	4.2 (0.6)	3.8 (0.2)
4 (Saeed)	1.9 (0.1)	4.3 (1.0)	5.0 (0.1)	1.4 (0.2)	3.9 (0.7)	3.5 (0.3)
Overall Mean	2.0 (0.2)	4.5 (1.1)	5.3 (0.5)	1.4 (0.2)	4.0 (0.7)	3.7 (0.4)

Note. M = Mean; SD = Standard Deviation. All means were calculated across observations within each phase for each participant.

## Data Availability

The original contributions presented in this study are included in the article. Further inquiries can be directed to the corresponding author.
